# Peek polymer in orthodontics: A pilot study on children

**DOI:** 10.4317/jced.54010

**Published:** 2017-10-01

**Authors:** Gaetano Ierardo, Valeria Luzzi, Michela Lesti, Iole Vozza, Orlando Brugnoletti, Antonella Polimeni, Maurizio Bossù

**Affiliations:** 1 Pediatric dentistry Unit, Department of Oral and Maxillo-facial Sciences, Sapienza University of Rome

## Abstract

The purpose of the study has been making the orthodontic space maintainers in PEEK polymer (Polyetheretherketone) through a digital workflow. New digital technologies are taking hold in diagnosis, therapy and in dental laboratories. The digital world can help dentist in diagnosis and therapy: -diagnosis through the acquisition of radiographic images (CBCT) or scanners which allow the creation of 3D digital models – about therapy thanks to dental CAD CAM system. It consists of design devices through an dedicated software CAD and then realize devices through CAM system. We used digital system to improve the quality of the treatment and reduce costs. Peek polymer, subject of studies in recent years, thanking to its characteristics, resulted useful for this study. According to a nine month- follow up the devices were found comfortable, satisfying, personalized and minimally visible for the patients. These devices were found suitable to maintain the space. About material, thanks to its dimensional stability, mechanical strength but specially, thanks to its biocompatibility, PEEK was found as a very good material to build space maintainers. The workflow allowed a simulation of the treatment plan with a better collaboration and acceptance of the patient. Digital system reduced the systematic mistakes during the various phases and the production time. The digital system saved space creating a virtual plaster casts collection.

** Key words:**PEEK, CAD/CAM system, space maintainers, orthodontic prevention.

## Introduction

Digital technologies and new materials are becoming popular, getting better and changing the way to do diagnosis and therapy in orthodontics (1). Polyetheretherketone (PEEK) is a semi-crystalline polymer commercialized from 1978 and composed by repeating units of three phenyl rings, two ester groups and one keto group (2). PEEK is characterized by excellent mechanical proprieties maintained to high temperatures. It is a rigid opaque material with a unique combination of properties, which include exceptional chemical, wear, electrical and temperature resistance, as well as dimensional stability and many processing capabilities (3). PEEK polymer is used typically as a replacement for machined metals in a wide variety of high performance and applications from components for cars, aircraft, industrial pumps, valves and seals, to silicon wafer carriers, connectors and serializable surgical instruments and in the medical implants market (4). This biocompatible material was used in recent years in the field of dental implantology because presents an elastic modulus of 3.6 GPa which is closer to the bone one (5). Peek is a certifiable material from the nutritional point of view responding to both the European and US legislation (FDA). Peek is employed in biomedicine, petroleum, chemistry and aerospace. A part from the chemical and mechanical properties, biocompatibility is the most important property for clinical use. Biomaterial is a material that can be used for a long time in contact with body tissues without causing rejection reactions. For a material to be biocompatible is must be non-toxic, non-immunogenic, non-mutagenic and non-carcinogenic. PEEK shows a natural tooth-colored appearance as compared to metal reconstructions (6). There has been no evidence that PEEK in bulk form fails any of the criteria for biocompatibility. By the late 1990s, PEEK thanks to its high performance thermoplastic is a candidate for replacing metal implant components, especially in orthopedics and trauma (7). Peek polymer was also studied and used in orthopedics for total knee replacement (8). Polyetheretherketone in recent years is the subject of several studies in which it was compared with other materials. Stawarczyk B. *et al.* studied the evaluation of the adhesion between PEEK and two self-adhesive resin cements after plasma treatment and the result has been the use of methyl methacrylate (MMA)-based adhesives allows bonding between PEEK and self-adhesive resin cements. Plasma treatment has no impact on bond to resin cements (9). As regards orthodontics, thanks to a study conducted in 2015 showed that the peek has many advantageous properties that make it a suitable candidate to use it as an esthetic metal-free orthodontic wire (10). This material can be used for patients allergic to metals, or who dislike the metallic taste or weight. Another study about a modified PEEK material, known as BioHPP, resulted to be a biocompatible, nonallergic, rigid material, with flexibility comparable to bone, good wear resistance, high polishing and low absorption properties and above all low plaque affinity (11). The aim of the study was first to test on children design and function of orthodontic space maintainers in PEEK polymer through a digital workflow splitted in the acquisition of 3D images (used for diagnosis and treatment planning), and the design of the devices using dedicated software (CAD) and their realization by milling (CAM). Secondarily the other aim was to check the function of the peek devices in order to favor the eruption of permanent teeth thanking the space maintenance in the arch.

## Case Report

The study took place in Pediatric Dentistry Unit, Department of Oral and Maxillofacial Sciences, “Sapienza” University of Rome, and began with the enrollment of 8-10 year old patients who needed space maintainers because of early loss of teeth for caries or extraction due to supernumerary tooth or abnormal inclination of permanent teeth. We made three prototypes of orthodontic devices: lingual arch, band with loop and removable plate. These devices had the purpose to maintain the space in the mouth of children during the phase of dental commute helping the correct transition from deciduous teeth to permanent teeth either in patients with deciduous decayed teeth or in patients subjected to extractions in orthodontic purpose (12). We used CAD/CAM system, a technology began its dental life in 1970s (13). The workflow has been divided into several steps:

Step 1: After the enrollment of patients, we took the dental precision impression, we poured models and then we digitalized models with a scanner in order to make a personalized appliance without standard measurements. We used an extraoral scanner (D810, 3Shape, Denmark). The scanned object was hit from all sides by light beams and then filmed with micro cameras. Since the scans are several and detected over the entire model the result was a cloud of points. The software connected the points and reconstructed a pattern of tiny polygons creating the virtual model.

**Figure 1:**
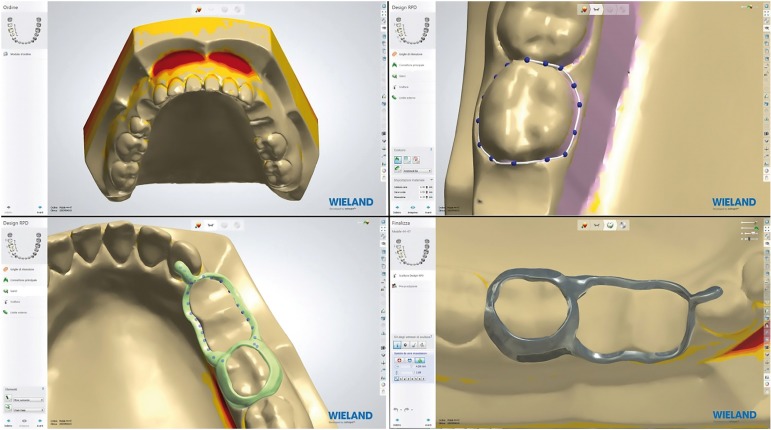
Digital pattern acquisition Thanks to CAD software and design of the devices.

Step 2: Once got virtual model, we had the model in all the screenings. Thanks to CAD (Computer Aided Design) software we designed personalized devices. The model has been archived and imported directly into 3Shape Dental Design software system using the zoom tools, rotating and panning allowed to view the model from different angles and magnifications facilitating the analysis of the model. This system allows to design devices and to determinate a lot of variables such as the material thickness, retention, undercuts, the space for the cementation, the points of support (Fig. 1).

Step 3: At this point the file has been sent to the CAM and begun the construction of the building through milling Roland DWX-50 features 5-axis continuous movement, equipped with an automatic transmission of different tools. This is a manufacturing process by subtraction and thanks to these movements the block of chosen material was milled to get the form designed previously by software CAD (in about one hour). The three devices we made, were the following: lingual arch, band with loop and removable plate. The first patient was 8-year old child who needed a space maintainers to keep the space in the mandible o allow a proper eruption of the canines and premolar teeth.

**Figure 2:**
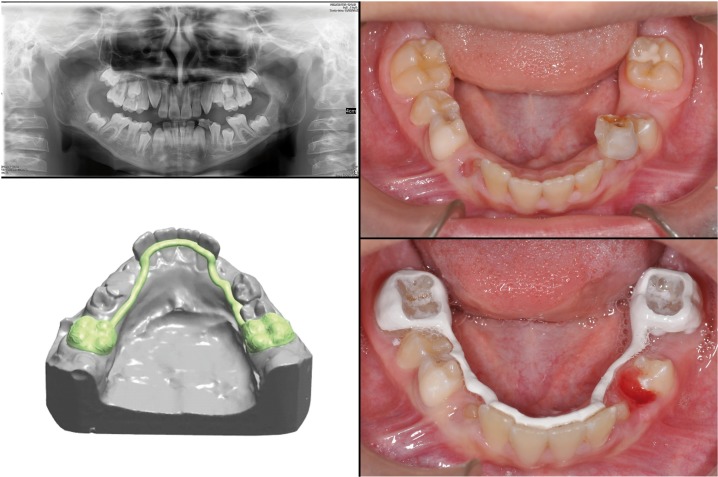
First case report.

**Figure 3:**
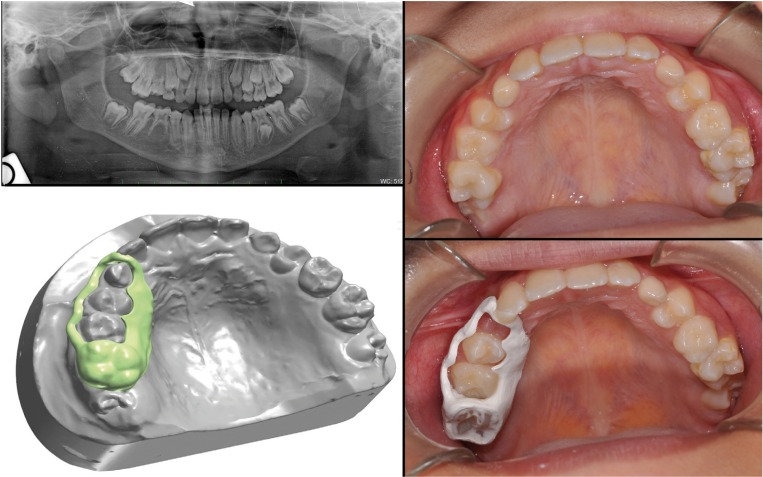
Second case report.

The workflow allowed us to get a peek polymer 1.3mm thick lingual arch, in about one hour, which could get in touch with facial lingual. The device was also composed by two orthodontic bands cemented on the first lower molars ( by cvi) (Fig. 2). In the second case, the planned device was a band with loop. The patient was ten years old and had an abnormal inclination of the permanent right upper canine. After clinical and radiographic evaluation we decided to extract the deciduous canine to favor the spontaneous eruption of the permanent canine. It was necessary a band on the first right molar and a loop in contact with the lateral right incisors in order to maintain the necessary space (Fig. 3).

**Figure 4:**
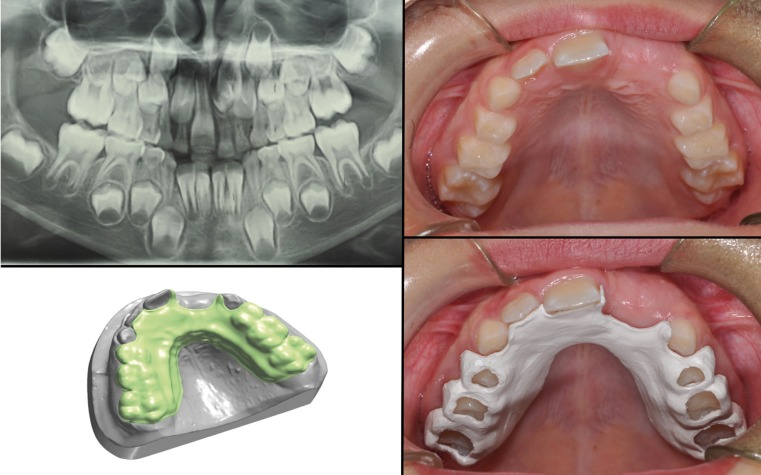
Third case report.

The last case shows a removable plate. The patient was 8 years old, had a supernumerary which interfered with the proper central incisor eruption. The supernumerary caused a delay of incisor eruption. Thanks to the central incisor’s eruptive force, the treatment plan was to extract the deciduous incisor and the supernumerary and to maintain the space during the permanent incisor’s eruption (Fig. 4). According to a nine month- follow up all 3 patients found the devices comfortable and very satisfying because they were personalized and minimally visible. These devices were found suitable to maintain the space. Then they remained stable, no discementation or fracture was observed. No allergy or presence of plaque was described. Thanks to its smooth surface the devices were well accepted by all little patients also because they were easy to polish and clean. Digital design of the appliance allowed to have better little patients compliance. About material, thanks to its dimensional stability, mechanical strength but specially thanks to its biocompatibility, PEEK was found as a very good material to build space maintainers. The workflow allowed a simulation of the treatment plan with a better collaboration and acceptance of the patient. Digital system reduced the systematic mistakes during the various phases, decreasing production time. It needs to stress the concept of digital and not hand-made steps in order to have greater precision and less discomfort. The digital system saved space creating a virtual plaster casts collection.

## Discussion

New research in dentistry such as digital technologies or tissue engineering are changing diagnosis and therapy (14). Digital technologies can also improve patient compliance and therefore the acceptance of therapies. In our pediatric dentistry Unit were made evaluations of customer satisfaction in order to improve the quality of service (15). In order to satisfy the research of new materials and new technologies we chose PEEK as material for space maintainers. The material chosen for this study was used in recent years in the field of dental implantology because presents an elastic modulus of 3.6 GPa which is closer to the bone one (5). By the late 1990s, PEEK thanks to its high thermoplastic performance is a candidate for replacing metal implant components, especially in orthopedics and trauma (7). Peek polymer was also studied and used in orthopedics for total knee replacement (8). Polyetheretherketone in recent years is the subject of several studies where it was compared with other materials (8,9). As regards orthodontics, Maekawa M. *et al.* showed that peek has many advantageous properties useful for esthetic metal-free orthodontic wire despite lower esthetic properties (10). There are no further clinical studies about using of peek polymer in orthodontics. The recent development and commercialization of colored PEEK promises significant improvement in this esthetic deficit hopefully next years clinical studies about this material will increase especially in medicine due to its advantages. From our prototypes analysis we can conclude that design and function of orthodontic space maintainers in PEEK polymer through a digital workflow has great potential as an alternative material for orthodontic appliances. Acquisition of 3D images, using of dedicated software (CAD) and milling (CAM) for manufacturing peek devices allowed to show the space maintenance in the arch in order to favor the eruption of permanent teeth and little patients compliance. Digital work is in constant evolution in dentistry. For this reason, we hope that more complex devices will be created with this method and also with this material in the future. These devices are prototypes and so these require a further clinical validations to replace traditional devices. Furthermore it’s necessary to consolidate the milling technique to improve the mechanical characteristics of the device. Other techniques such as 3D printing may be an alternative option that can be studied. Further clinical studies are also necessary to establish their active function once defined the passive role of these devices.
